# Inflammatory myofibroblastic tumours: a pictorial review

**DOI:** 10.1007/s13244-014-0370-0

**Published:** 2014-12-18

**Authors:** Jon Etxano Cantera, María Páramo Alfaro, David Cano Rafart, Romina Zalazar, Maite Millor Muruzabal, Paula García Barquín, Isabel Vivas Pérez

**Affiliations:** 1 Grupo Hospitalario Quirón, Carretera de Leioa-Unbe, 33 bis, 48950 Erandio, Vizcaya Spain; 2 Clínica Universidad de Navarra, Avenida Pío XII 36, 31008 Pamplona, Navarra Spain

**Keywords:** Inflammatory myofibroblastic tumours, Ultrasonography, Computed tomography, Magnetic resonance

## Abstract

**Objectives:**

To present the most important characteristics of inflammatory myofibroblastic tumours (IMTs) arising in different locations of the body with histological correlation.

**Methods:**

To review the symptoms and main radiological findings of IMTs. On ultrasonography (US), these tumours can appear as hypoechoic or hyperechoic masses and a variable Doppler appearance with increased vascularity. Computed tomography (CT) and magnetic resonance (MR) are the most used imaging tools in their evaluation. On contrast-enhanced CT, IMTs can appear as homogeneous or heterogeneous lesions, with variable enhancement on delayed acquisitions due to fibrosis. These findings are also present on gadolinium contrast-enhanced MR. On T1-weighted and T2-weighted sequences, IMTs usually show low signal intensity reflecting also the presence of fibrotic tissue.

**Results:**

To show the main clinical symptoms and radiological features of IMTs in different locations: head and neck, lung, genitourinary, hepatic, splenic, gastrointestinal tract, mesenteric, muskuloskeletal.

**Conclusions:**

Although IMTs in some organs are not uncommon, they are not usually included in the differential diagnosis of masses. Their radiological features suggest malignant neoplasms, whereas they are not. Consequently, this is an underdiagnosed entity and only after an histological exam could a definitive diagnosis be achieved.

***Teaching Points*:**

• *Their radiological features suggest malignant neoplasms, whereas they are not*

• *CT and MR imaging are the most used tools in their evaluation*

• *IMT is an underdiagnosed entity*

• *The definitive diagnosis is only after histological exam*

## Introduction

Inflammatory myofibroblastic tumours (IMTs) constitute a rare group of neoplasms composed of a mixture of spindle-shaped myofibroblasts or fibroblasts and a variable amount of inflammatory cells (eosinophils, plasma cells and lymphocytes) [1]. Many different terms have been used to refer to these tumours: plasma cell granuloma, inflammatory myofibrohistiocytic proliferation, fibroxanthoma, xanthogranuloma. However, nowadays IMT is the most accepted. The most frequently affected organs are lung and orbit [2], but they have been described in nearly every organ.

Different aetiologies have been proposed for IMT [3], being different chronic infections, autoimmune diseases and trauma the most accepted. Specific inflammatory diseases, such as IgG4 disease, have also been recently associated [4, 5]. Little information exists about the natural history of this entity. In some cases, an aggressive behaviour with metastases has been described [3, 6, 7].

Clinical presentation of IMTs depends on the organ in which they arise, but they frequently associate general inflammatory symptoms as fever or malaise.

Radiological appearance of IMTs is unspecific and they are often misdiagnosed as malignant neoplasms. Many of them are incidentally discovered when an imaging technique (computed tomography [CT], ultrasonography [US] or magnetic resonance imaging [MRI]) is performed for any other reason. Their most common radiological presentation is as solid, irregular, well-defined masses.

Histological studies are critical to properly diagnose them. Immunohistochemical studies of T- and B-cell subpopulations, CD34, CD117, S-100 and c-Kit may be helpful in distinguishing IMTs from other soft-tissue neoplasms [8, 9]. At the molecular level, approximately half of IMTs contain a clonal cytogenetic aberration that activates the anaplastic lymphoma kinase (ALK-) [9]. Positive immunohistochemical staining of ALK is in approximately 40–100 % of IMTs, depending on the anatomical sites where they arise [8–10]. This finding suggests a possible neoplastic cause of IMTs. Furthermore, ALK expression could be a prognostic factor of aggressiveness for IMT.

According to an update based on the new World Health Organisation (WHO) classification, IMTs are considered as neoplasms which may recur or metastasise in as many as 5 % of cases [11].

The objective of this pictorial review is to present the most important characteristics of IMTs arising in different locations of the body with their histological correlation.

## Imaging techniques

The radiological findings of IMTs are non-specific. On US, these tumours can appear as hypoechoic or hyperechoic masses with ill-defined or well-circumscribed edges and a variable Doppler appearance with increased vascularity. US is usually the first imaging technique when IMTs appear in specific locations, such as testicles or neck. In other locations its role is limited except for the guidance of percutaneous biopsies.

CT and MRI are the most used imaging tools in the evaluation of IMTs. On contrast-enhanced CT, IMTs can appear as homogeneous or heterogeneous lesions, with variable enhancement on delayed acquisitions due to the presence of fibrosis. These findings are also present on gadolinium contrast-enhanced MRI. On T1-weighted and T2-weighted sequences, IMTs usually show low signal intensity reflecting also the presence of fibrotic tissue (Table 1).

**Table 1:** Symptoms, radiological findings and differential diagnosis of the main IMTs of the body

	Symptoms	Imaging findings	Differential diagnosis
Orbit	Pain, redness, oedema, proptosis, ptosis, oculomotor deficits, diplopia, swelling, mass-effect	Solid and heterogeneous enhancing. Low intensity on T2-weighted due to fibrotic composition. Associated retrobulbar fat or oedema	Granulomatous diseases, primary infection, sarcoid, Sjögren disease, primary and secondary tumours of the orbit, lymphoma and connective tissue diseases
Lung	Cough, chest pain, dyspnea, haemoptysis, fever, malaise and weight loss.	Solitary, well-circumscribed, peripheral, predominance for the lower lobes, unusual calcifications. Heterogeneous or homogeneous enhancement pattern. Low intensity on T2-weighted images	Malignant lung masses: primary bronchogenic carcinoma
Scrotum	Lump in the scrotum	Solid and heterogeneous masses with internal vascularity on US. Well-defined, hypodense, little enhancement on CT. Small calcifications can be associated	Scrotum primary neoplasm
Hepatic	Unspecific, depending on the size	Heterogeneous or peripheral enhancement during the arterial phase. Or homogeneous enhancement during the arterial phase and washout during the delayed phase on CT. T1 hypointense and T2 hyperintense with heterogeneous enhancement	HCC
Splenic	Left upper quadrant or epigastric pain	Low-density pattern. Low-intensity on the T1- and T2-weighted images. Low-intensity lesion in the early phase after gadolinium injection and high-intensity in the delayed phase	Splenic haemangioma and other primary splenic neoplasms as lymphoma or splenic angiosarcoma
Grastrointestinal tract and mesentery	Abdominal pain, intestinal obstruction, dysphagia, anaemia	Hypodense, heterogeneous, well-delimited	GIST, inflammatory fibroid polyp, smooth muscle neoplasm, peripheral nerve sheath tumour, solitary fibrous tumour, fibromatosis, the follicular dendritic cell sarcoma, lymphoma and adenocarcinoma
Musculoskeletal	Pain, oedema	Non-homogeneous, solid	Malignant lesions: rabdomyosarcoma

## IMTs in different locations of the body

### Head and neck IMTs

**Fig. 1:**
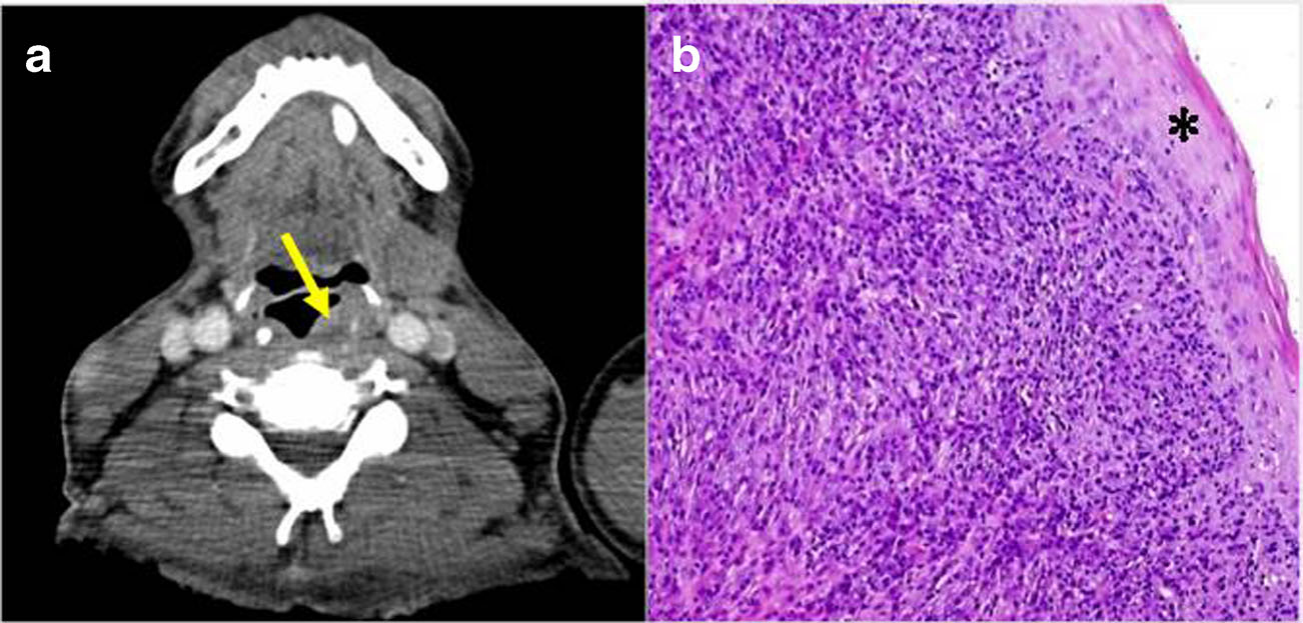
PATHOLOGY: IMT in the left supraglottic space (*arrow*) with paraglottic space involvement indicated by replacement of the paraglottic fat with soft tissue. **a** Axial reformation of contrast-enhanced neck CT. A solid, well-defined, little-enhancing nodule in the supraglottic larynx was observed (*yellow arrow*). It did not seem to infiltrate adjacent structures. **b** Microscopic study obtained after surgical removal of the lesion. The sample showed proliferation of fusiform cells mixed with macrophages and giant multinucleated cells. Small number of atypia and mitotic figures were present. The tumour presented an expansive growth pattern. Normal tissue was present in the peripheral zone (*)

IMTs have been reported in various sites in the head and neck such as bucal space, maxillary sinus, nasal cavity, parotid gland, nasopharynx and larynx (Fig. 1).

However, the orbit is the most common location of IMTs. They account for approximately 10 % of orbital masses, being the third most common cause of orbital masses [12]. Their aetiology is unknown, but they have been associated with inmunological disorders, different infections and history of trauma. Recently, they have been strongly associated with the IgG4-related sclerosing systemic inflammatory disease [13] and with chronic infection by Epstein-Barr virus [14].

The clinical presentation and symptoms may vary with the location, extension and aggressiveness of the tumour. Patients with IMTs of the orbit can present local symptoms as pain, redness, oedema, proptosis, ptosis, oculomotor deficits, diplopia, lid swelling or mass-effect and systemic symptoms due to the general inflammatory disorders they are frequently associated with [15].

CT and MRI are the imaging techniques of choice to evaluate these lesions. Both of them provide information about the exact location and extension of the mass. MRI is superior to CT in the evaluation of inflammation of the nerves and muscles [16].

**Fig. 2:**
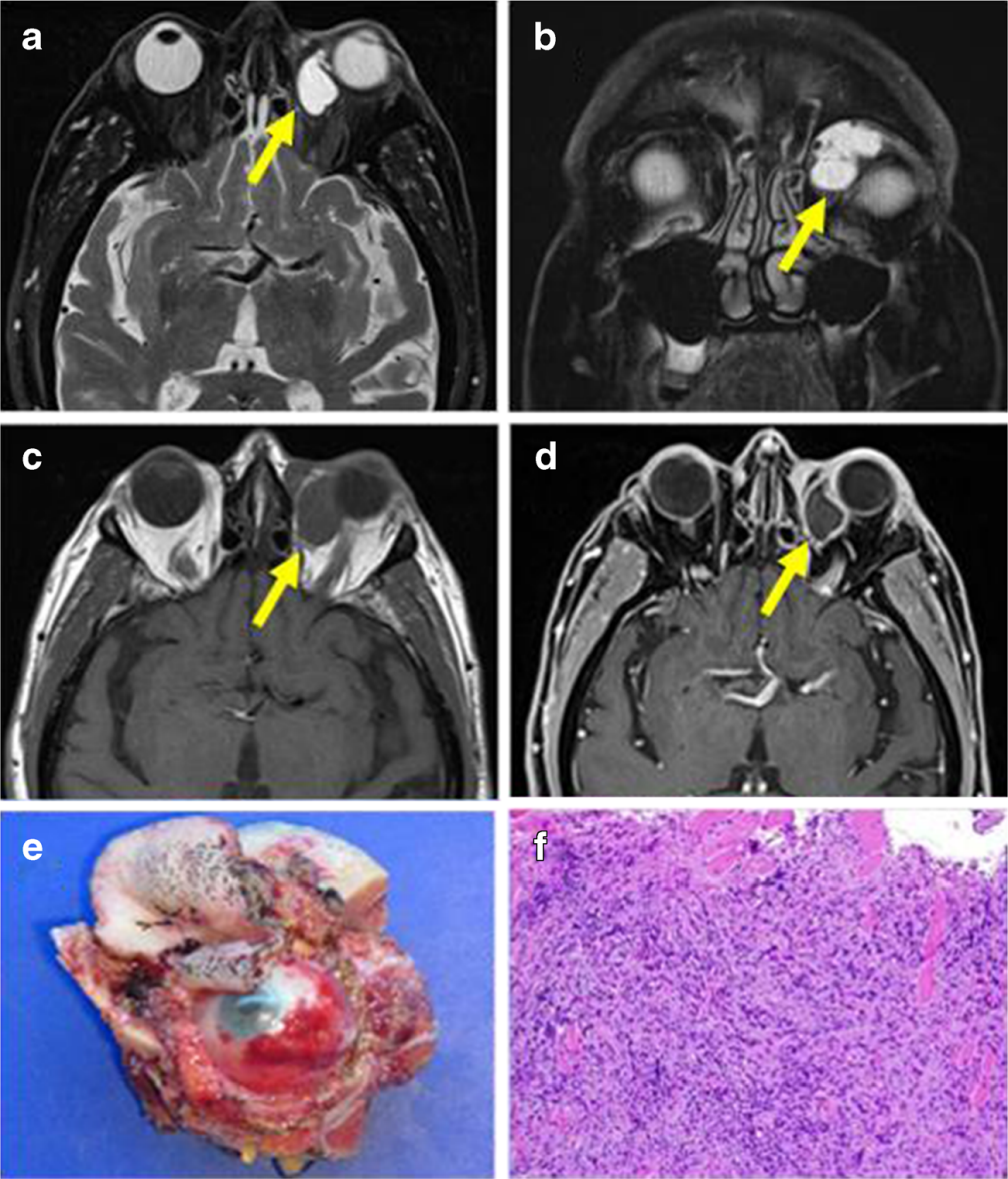
PATHOLOGY: IMT of the left orbit. **a**, **b** Axial and coronal MR reformations of the same patient on T2-weighted sequences showed a well-delimited, hyperintense intraorbital mass, in the intraconal compartment, in the medial aspect. **c**, **d** T1-weighted images demonstrated a hypointense, homogeneous, well-delimited mass with peripheral enhancement after gadolinium injection. **e** Surgical sample obtained after resection. **f** Microscopic sample obtained after surgery demonstrated an epithelial tumour composed of fusiform cells mixed with an extensive chronic inflammatory infiltrate of plasmatic cells, lymphocytes and macrophages. In the peripheral of the sample striated muscle cells were also observed

They usually appear as solid and heterogeneous enhancing masses, which can affect retro bulbar fat, cause bone destruction, and present intracranial extension [17]. When they show low intensity on T2-weighted images prominent, fibrotic composition is present (Fig. 2).

Pathologically, IMTs of the head and neck consist of many fibroblastic cells, fibroblastic cells and inflammatory cells, which include plasma cells and eosinophils.

The differential diagnosis includes granulomatous diseases, primary infection, sarcoid, Sjögren disease, primary and secondary tumours of the orbit, lymphoma and connective tissue diseases [16].

Treatment depends on its location and extension. The administration of corticosteroids an inmunosupresors usually leads to a decrease of size of the mass but also radiotherapy or surgery may be indicated.

### Pulmonary IMTs

Lung is one of the most common locations of IMTs. They account for around the 50 % of benign pulmonary masses in children, representing only about 0.7 % of all tumours of the lung in general population [18].

As in other organs, they are also associated with immunological disorders [18] and chronic infections [19]. Sometimes, IMTs are also related with antecedents of lung surgery and they can arise in surgical lung scars.

Common clinical characteristics include unspecific respiratory symptoms, such as cough, chest pain, dyspnea, haemoptysis and unspecific inflammatory symptoms as fever, malaise and weight loss.

**Fig. 3:**
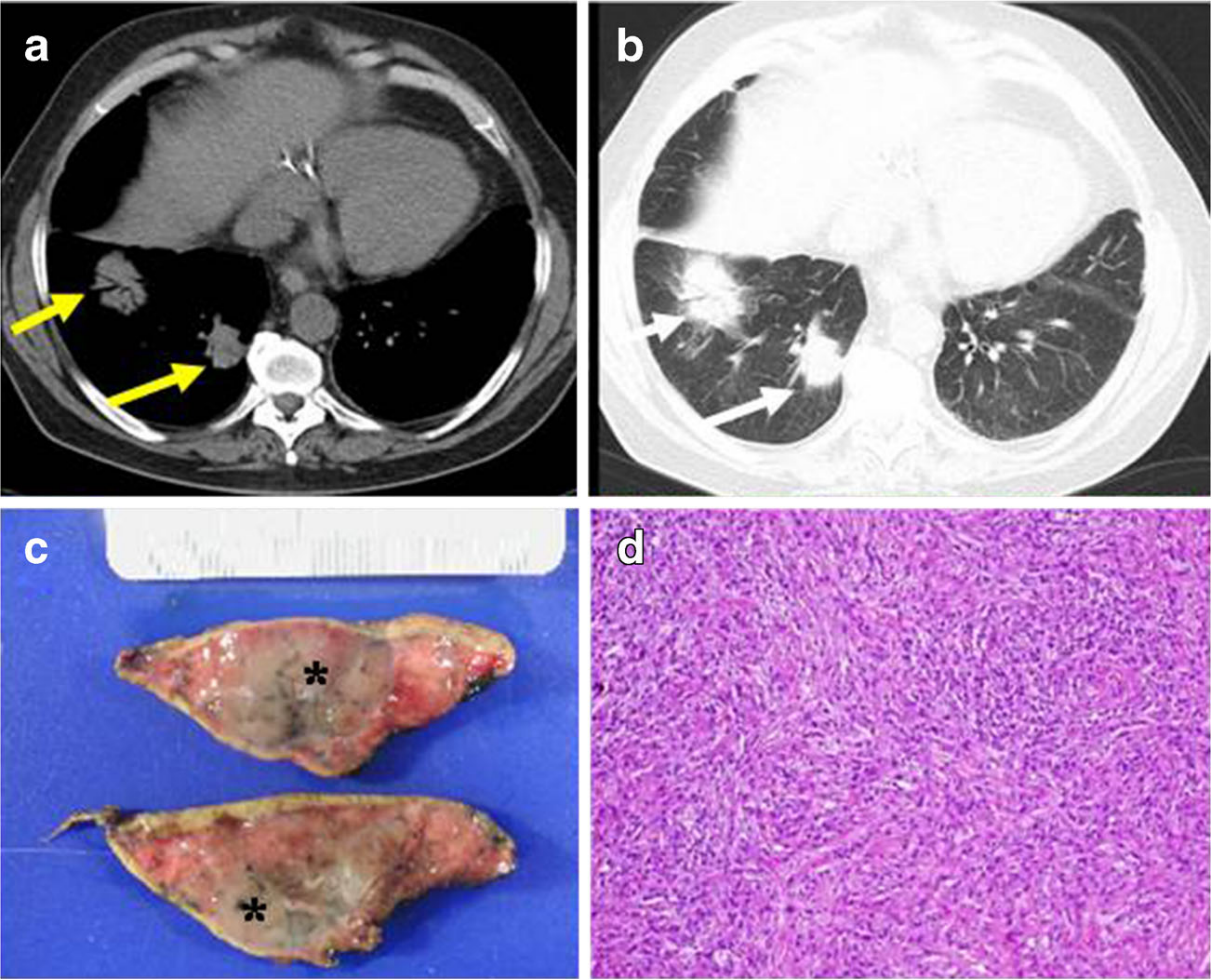
PATHOLOGY: IMT of the lung. A 55-year-old man with a cough and haemoptysis.**a** Axial reformation of non-contrast CT with mediastinum window. Two solid, ill-defined nodules in right lower lobe are observed (*yellow arrows*). Note the air brocogram observed in the biggest mass. **b** Axial reformations with lung window of the same patient where the lung nodules are shown (*white arrows*). **c** Partial lobectomy of the right lower lobe specimen. Note the presence of the nodules (*). **d** Microscopic study of the resected lung sample where fusiform cells with an associated inflammatory infiltrate of lymphocytes, plasmatic cells and histiocytes was found

The radiological presentation of lung IMTs is non-specific. They usually appear as solitary, well-circumscribed peripheral lung masses [20], with predominance for the lower lobes [21] (Fig. 3). Calcification of the masses is unusual (about 15 %) [22]. It is not frequent, but they can also be multiple. On CT with intravenous contrast, they present a variable heterogeneous or homogeneous degree of enhancement pattern [3, 21]. It has been described an aggressive behaviour with invasion of the adjacent structures [15]. On MRI, they present similar radiological findings as in other locations. Because of their similar radiological appearance to malignant lung masses, biopsy is necessary to obtain the diagnosis.

The treatment of choice of lung IMTs is surgery to exclude malignancy and to achieve the cure. Although spontaneous regression may occur, local expansion may cause significant morbidity and occasional death [23]. The prognosis after complete surgical resection is excellent, with a low rate of recurrence. In these cases, alternative treatments (radiotherapy, corticoids or chemotherapy) to surgery could be also used.

### Genitourinary IMTs

**Fig. 4:**
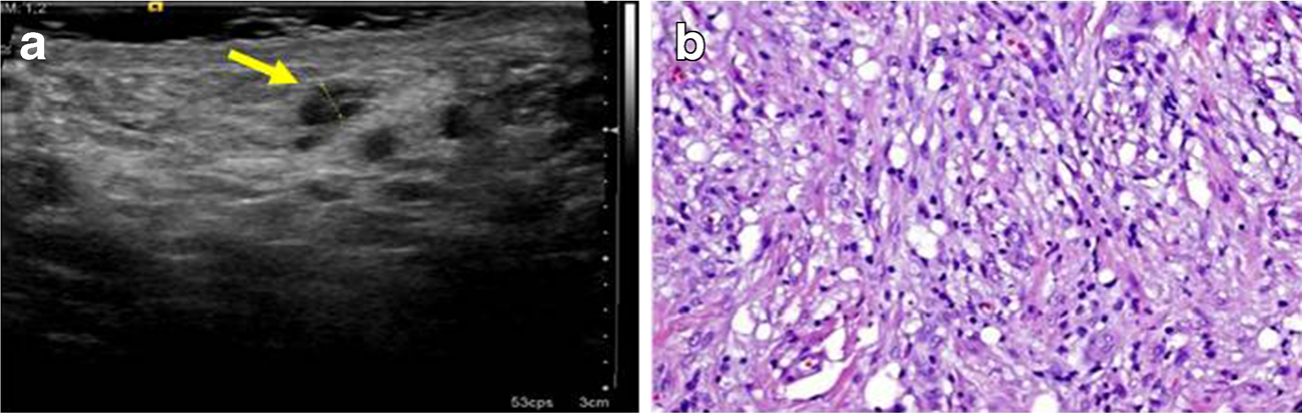
PATHOLOGY: IMT of the epididymus. A 40-year-old man with a lump in the scrotum. **a** A hypoechoic, well-delimited epididymal lesion in the right scrotum was detected. **b** Sample of the epididymal lesion obtained after surgical resection of the mass. A diffuse infiltrate composed of fibroblastic and fusiform cells mixed with inflammatory cells (mainly lymphocytes and macrophages) was observed

Only a few cases of IMTs in the spermatic cord have been reported. The most common presentation of epididymal (Fig. 4) and paratesticular IMTs is a lump in the scrotum.

**Fig. 5:**
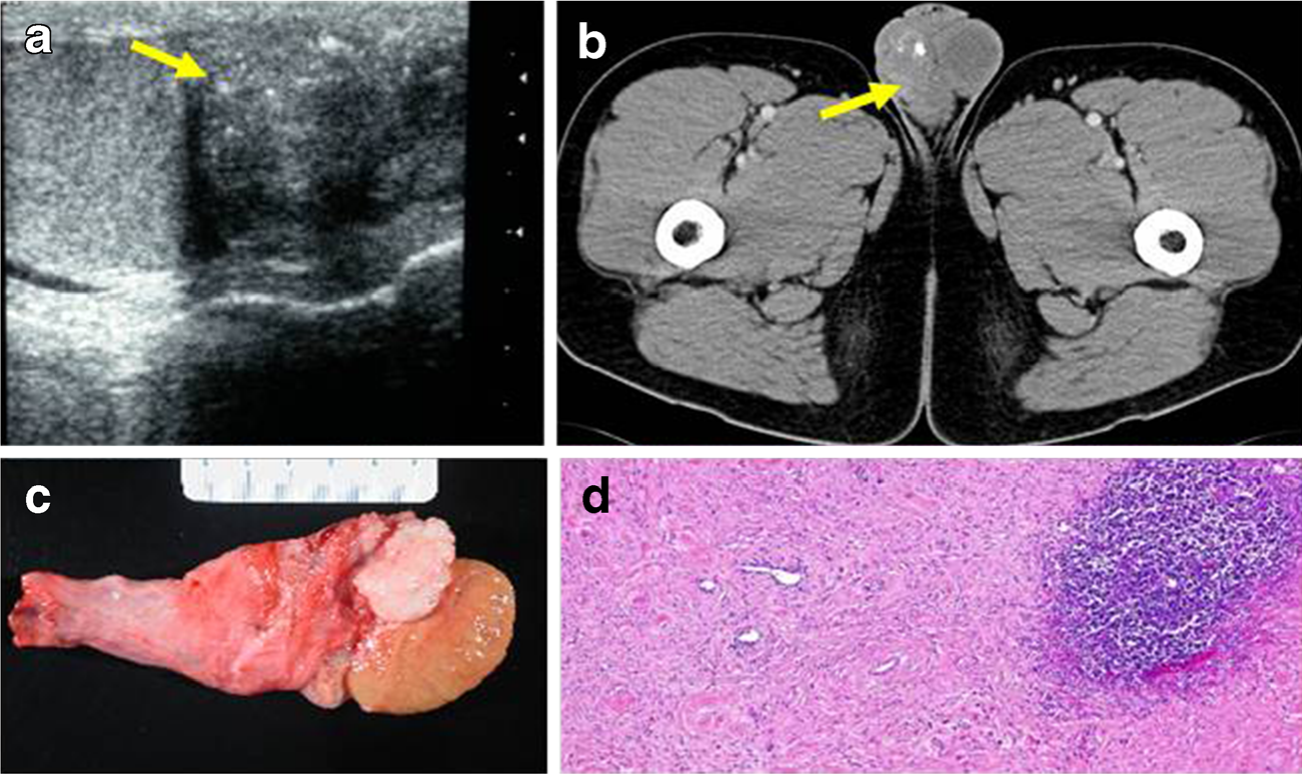
PATHOLOGY: paratesticular IMT. A 34-year-old man with no relevant medical history came to our hospital with a lump in the scrotum. **a** US showed an isoechoic, solid, paratesticular nodule with fine calcifications (*yellow arrow*). It did not seem to infiltrate the adjacent testicle. **b** Axial reformation of contrast-enhanced CT on portal phase demonstrated a well-delimited, heterogeneous, little-enhancing mass in the right scrotum. **c** Surgical sample obtained after orchiectomy. **d** Microscopic study of the lesion demonstrated a well-delimited lesion constituted of fibroblasts, lymphocytes and plasmatic cells with associated lymphoid follicle. There was no evidence of adjacent testicle invasion

In the evaluation of scrotal masses, US is the most accurate imaging technique, helping to distinguish intratesticular from extratesticular lesions and solid from cystic lesions. IMTs are described as solid and heterogeneous masses with internal vascularity [5]. On CT, they appear as well-defined, hypodense masses with little enhancement after intravenous contrast injection. In some cases, small calcifications can be associated (Fig. 5).

Surgical resection without orchiectomy is the ideal treatment, but in some cases the testicle must be removed due to the size of the mass or when the mass is attached to the testis.

**Fig. 6:**
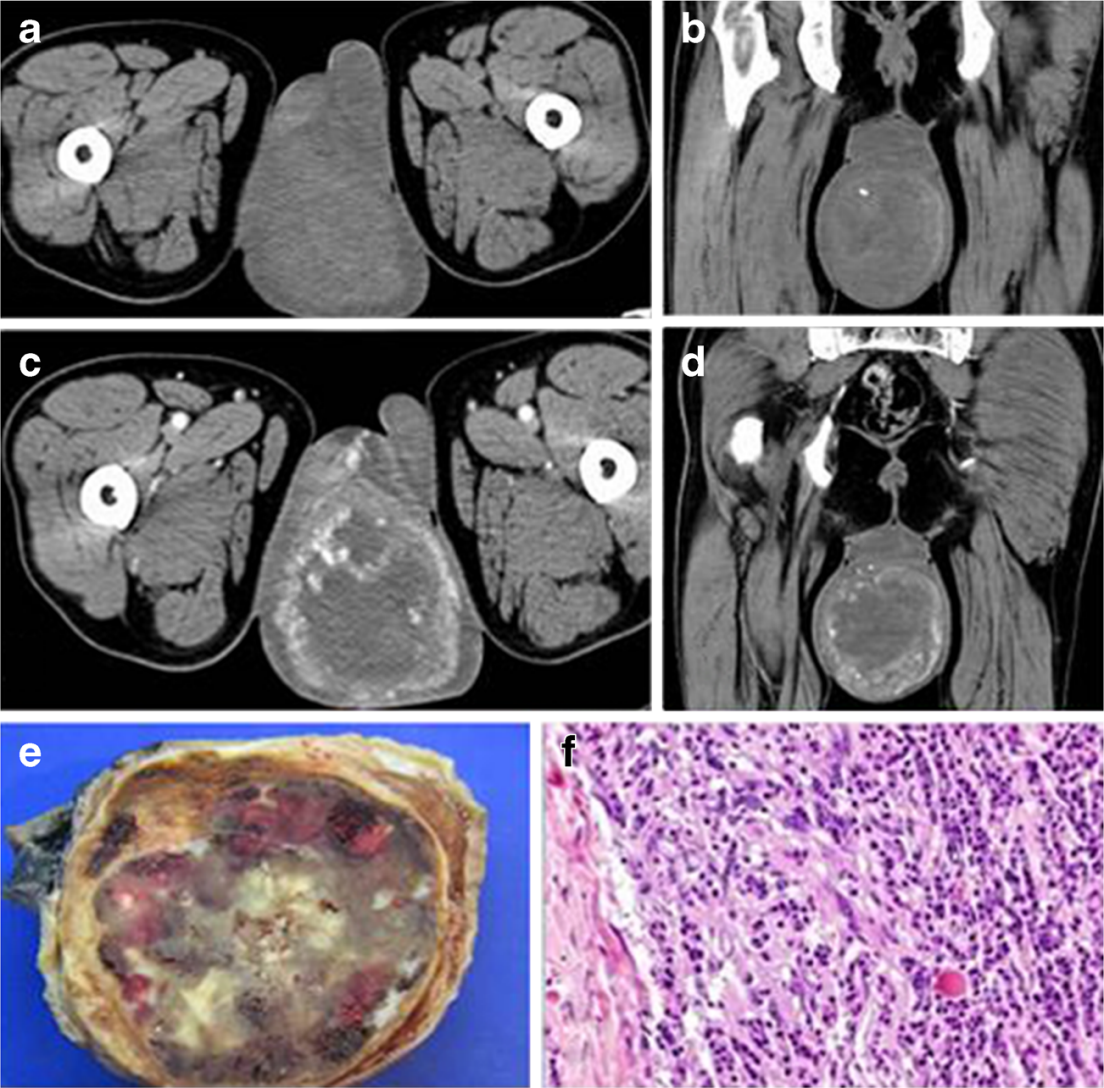
PATHOLOGY: IMT of the penis. A 46-year-old man with a mass in the penis came to our hospital. He related more than 6 years of slow growth of the mass with no suspicious associated symptoms. **a**, **b** Axial and coronal reformations of non-enhanced CT. Solid, well-defined, heterogeneous penile mass is shown. **c**, **d** Axial and coronal reformations of enhanced-CT after the injection of intravenous contrast on portal phase. The penile mass presents an heterogeneous and peripheral enhancement. **e** Surgical sample obtained after partial penectomy. Macroscopic view showed an heterogeneous aspect due to the mixture of tissues (solid tumour, lipoid component and areas of haemorrhage). **f** Microscopic study confirmed a mesenchymal tumour composed of fusiform cells, some of them multinucleated, with round or elongate nucleus. Prominent infiltrate of plasmatic cells and focal areas of haemorrhage were also found. No mitotic figures were observed

IMTs of the penis are extremely rare. To our knowledge, no IMTs at this location have been reported. We have recorded only one case in our institution (Fig. 6). On CT, this tumour appeared as a well-defined, little-enhancing and non-aggressive mass surrounding the penile urethra and the corpora cavernosa with no evidence of invasion of them.

Surgical treatment was performed due to the size and non-conclusive radiological features of the tumour. Histological studies confirmed the diagnosis of IMT.

### Hepatic IMTs

Hepatic IMTs constitute an extremely rare group of hepatic tumours, accounting only for the 0.7 % of all the hepatic lesions [24]. They are more frequent in young adults and Asian people. They have been associated with autoimmune diseases, immunological disorders and viral infections, such as IgG4 disease [25], sclerosing cholangitis [26], Crohn′s disease, hepatic viral infections [22] and Epstein-Barr virus [24].

Symptoms of hepatic IMTs depend on their size. Compression of adjacent hepatic structures has been described, producing obstructive jaundice. Weight loss and fever have also been reported [2].

**Fig. 7:**
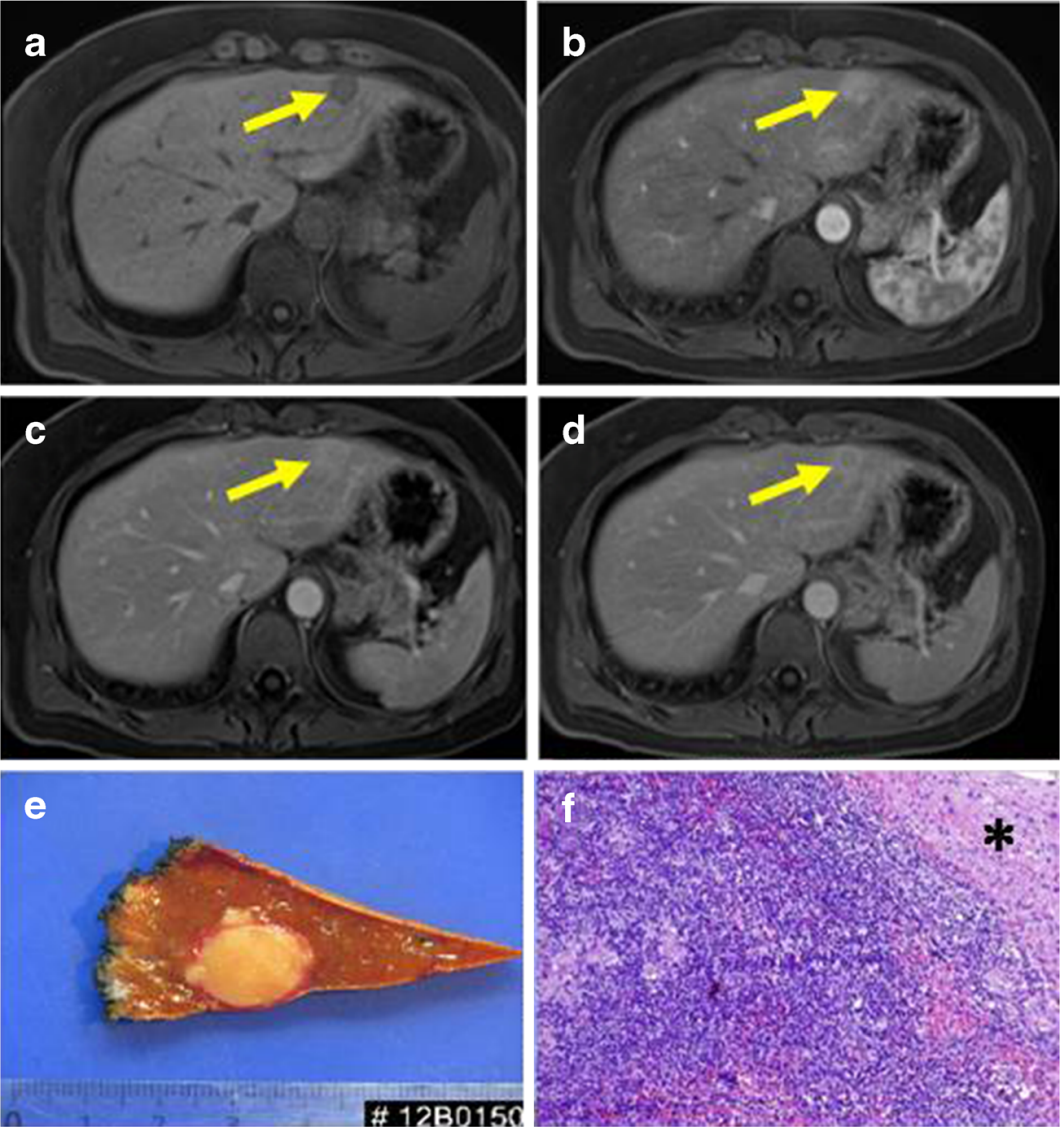
PATHOLOGY: IMT of the liver. Axial reformations of T1-weighted MR before and after intravenous gadolinium injection. Well-defined, hypointense lesion on T1-weighted images (**a**) which presented weak enhancement in the arterial phase (**b**) and wash-out on portal and equilibrium phases (**c**-, **d**) located in hepatic left lobe. Capsule was present. **e** Macroscopic view of partial hepatectomy obtained after surgical excision of the lesion. A solid, well-defined and yellowing appearance mass is showed. **f** The lesion demostrated an expansive chronic inflammatory infiltrate of plasmatic cells, lymphocytes and macrophages. The nearby hepatocytes (*) were normal

**Fig. 8:**
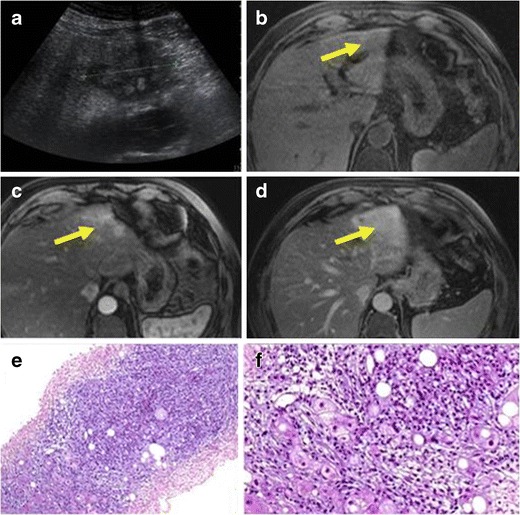
PATHOLOGY: hepatic IMT. **a** Abdominal ultrasound where a slightly hypoechoic, solid, heterogeneous mass arising in the left hepatic lobe was observed. Axial T1-weighted unenhanced (**b**) and contrast-enhanced MR images (**c**, **d**) showed an ill-defined with heterogeneous enhancement mass located in lateral hepatic segments. **e**, **f** Histological sample obtained after percutaneous biopsy. Mesenchymal tumour composed of fusiform cells, lymphocytes and lipid-filled macrophages can be observed. Normal hepatocytes with large and small fatty droplet change are also present

CT and MRI are the most used imaging techniques. Hepatic IMTs present different enhancement patterns according to their vascularity. On contrast-enhanced CT, they usually show heterogeneous or peripheral enhancement during the arterial phase. Despite this, some of them present homogeneous enhancement during the arterial phase and washout during the delayed phase, which can lead to misdiagnosing them as hepatocarcinomas (HCCs) [27] (Fig. 7). On MRI, these lesions usually are T1 hypointense and T2 hyperintense with heterogeneous enhancement (Fig. 8). Hepatobiliary contrast (Gadoxetate disodium, Primovist; Bayer Healthcare, Leverkusen, Germany) could be of help to differentiate HCC from IMTs of the liver. On the hepatobiliary phase, high signal intensity in the centre of the lesion has been described [27], suggesting a benign lesion.

Surgery is the most accepted treatment. If there is a biopsy-proven diagnosis of IMTs that excludes malignancy, medical treatment with no steroidal anti-inflammatory drugs in patients with peripheral hepatic IMTs could be prescribed [28].

### Splenic IMTs

Splenic IMTs are extremely rare. As in hepatic IMTs, Epstein-Barr virus has been proposed as a possible aetiological agent.

Patients with spleen IMTs frequently associate unspecific symptoms as left upper quadrant or epigastric pain. Splenomegaly is common and some patients can present fever, anaemia and signs of hypersplenism [29].

**Fig. 9:**
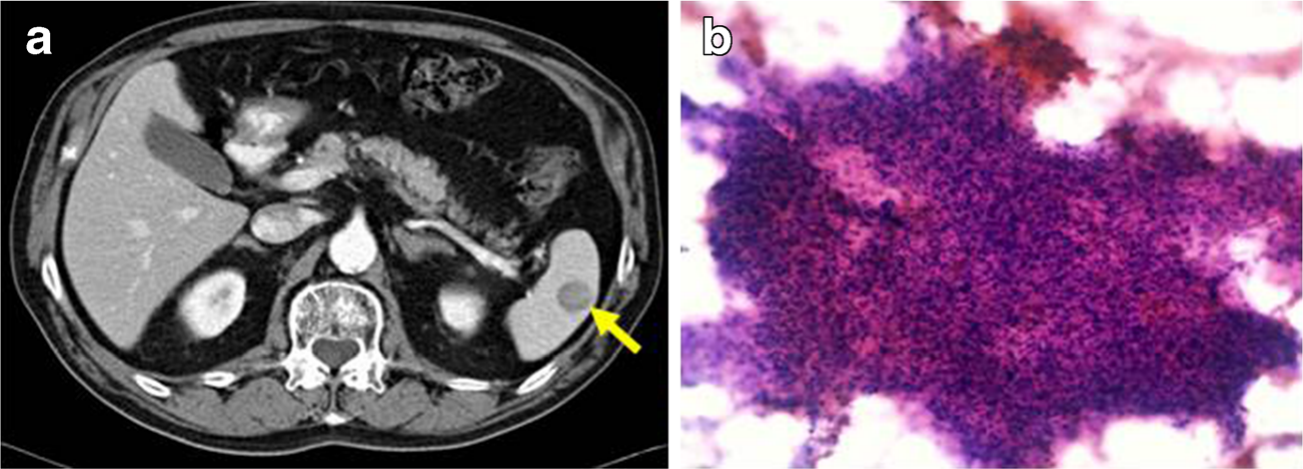
PATHOLOGY: IMT of the spleen. **a** Axial reformation of contrast-enhanced CT on portal phase. Solid and well-defined lesion, hypodense to the surrounding spleen parenchyma. **b** Sample obtained with fine-needle aspiration. Prominent cellularity composed of an irregular, weakly basophil set of mioepithelial-fusiform cells. Numerous inflammatory cells (plasmatic, lymphocytes and few neutrophils and eosinophils) with isolated macrophages were also found. No atypia or mitotic figures were present

Different radiological imaging modalities have been proposed for their evaluation. On all of them, the most common radiological finding is a well-delimited mass. On US, they look like echogenic or hypoechoic masses [30]. On CT, they are depicted as low-density masses in both non-enhanced and enhanced acquisitions (Fig. 9). On MRI studies, they present low-intensity mass on both the T1- and T2-weighted images. Their typical enhancement pattern after gadolinium injection is as a low-intensity lesion in the early phase, which changes into a high-intensity one in the delayed phase [29].

The differential diagnosis includes splenic haemangioma and other primary splenic neoplasms such as lymphoma or splenic angiosarcoma [30].

When a suspicious spleen mass is found, the treatment of choice is splenectomy due to the high risk of bleeding associated to splenic biopsy [8, 31]. The prognosis of this entity after splenectomy has been considered excellent [29].

### Gastrointestinal tract IMTs

IMTs of the gastrointestinal tract are infrequent. They can arise anywhere, the stomach and small intestine being the most common locations [3, 9].

Clinical symptoms may vary from none to abdominal pain, intestinal obstruction, dysphagia or anaemia, depending on the location and size of the mass. They also may present fever and malaise.

**Fig. 10:**
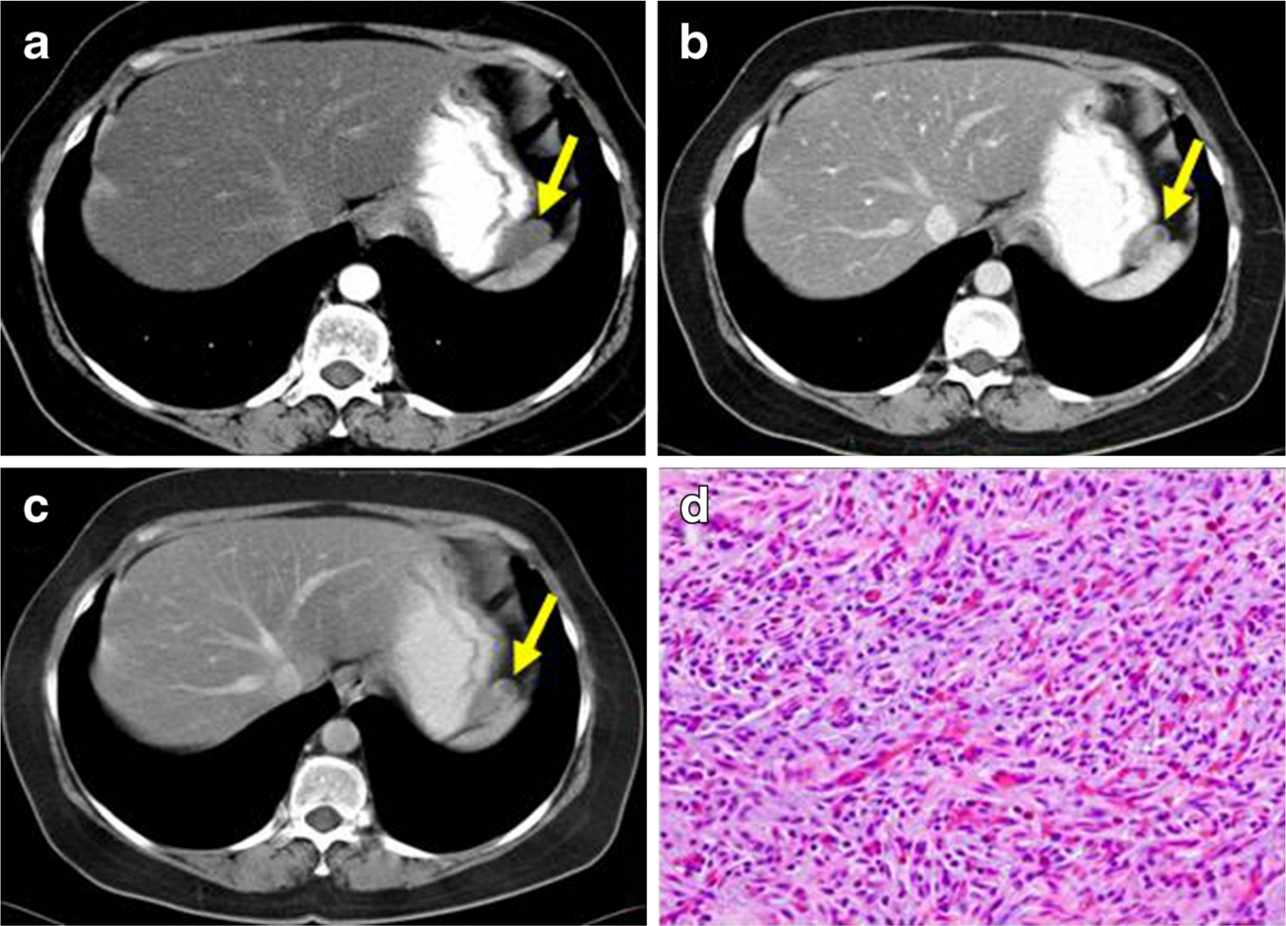
PATHOLOGY: IMT of the stomach. Axial reformation of contrast-enhanced CT on arterial (**a**), portal (**b**) and 3 min after injection delayed (**c**) acquisitions. A well-defined, heterogeneous nodule with moderate enhancement on portal phase arising from the gastric fundus was found (*yellow arrows*). Diffuse liver steatosis can also be observed. **d** Microscopic studies performed after surgical removal of the lesion demonstrated high amount of mesenchymal fusiform cells combined with vascular structures. Small focis of inflammatory infiltrates were present. The lesion showed well-defined contours and an expansive growth pattern

**Fig. 11:**
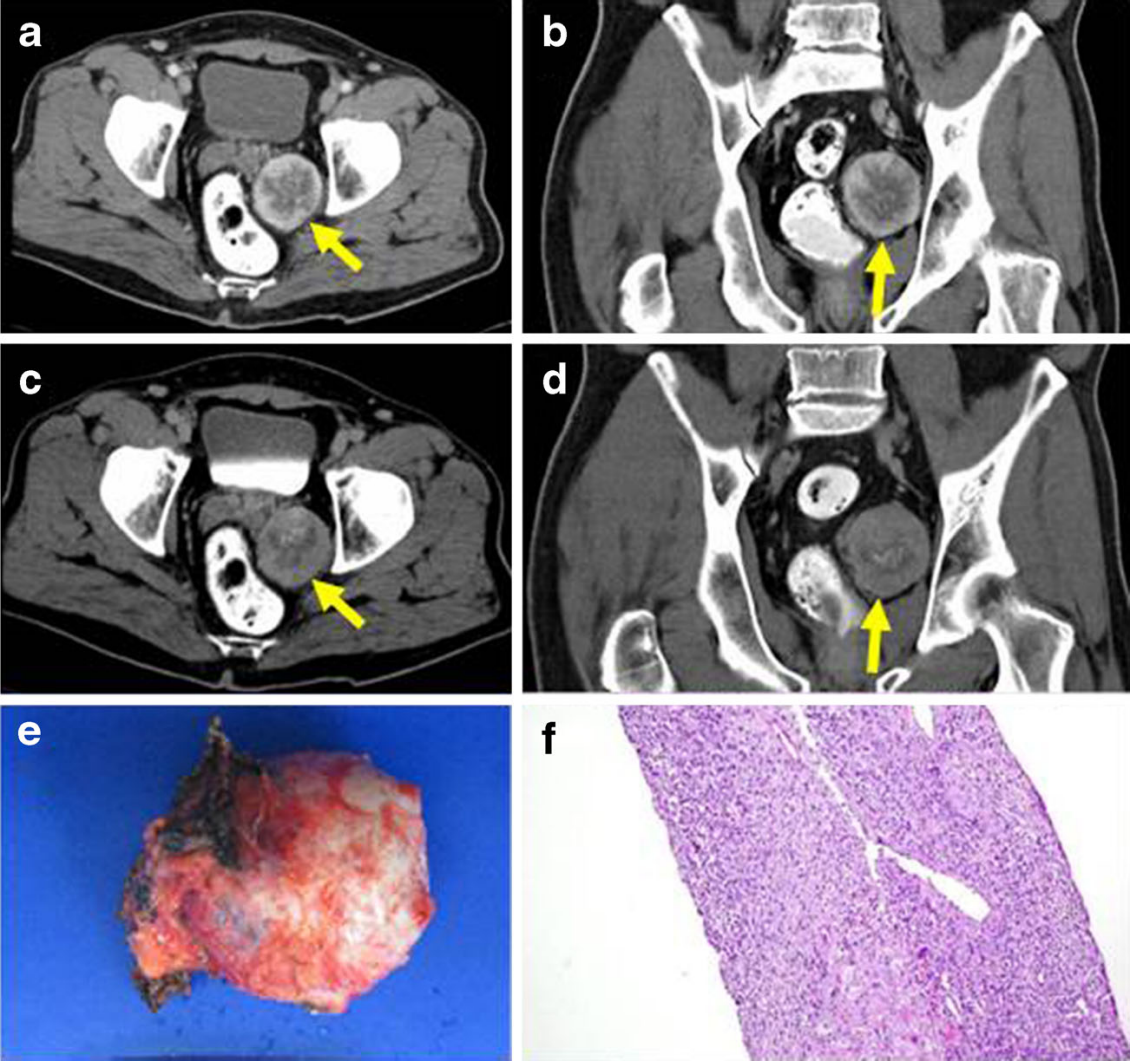
PATHOLOGY: pararectal IMT. Axial and coronal reformations of contrast-enhanced CT on portal (**a**, **b**) and 8 min after injection delayed acquisitions (**c**, **d**). Pararectal solid and well-defined mass with moderated peripheral enhancement adjacent to the rectum and left seminal vesicle was detected. **e** Macroscopic view of the tumour after surgical resection. **f** Histological sample obtained after biopsy. Mesenchymal neoplasm composed of polygonal cells with clear cytoplasm and round normochromatic nuclei with abundant vessels and occasional scattered inflammatory cells was observed. Mixed within the neoplasm cells, there was a chronic inflammatory infiltrate of lymphoid cells. There were no foci of necrosis or mitotic figures

**Fig. 12:**
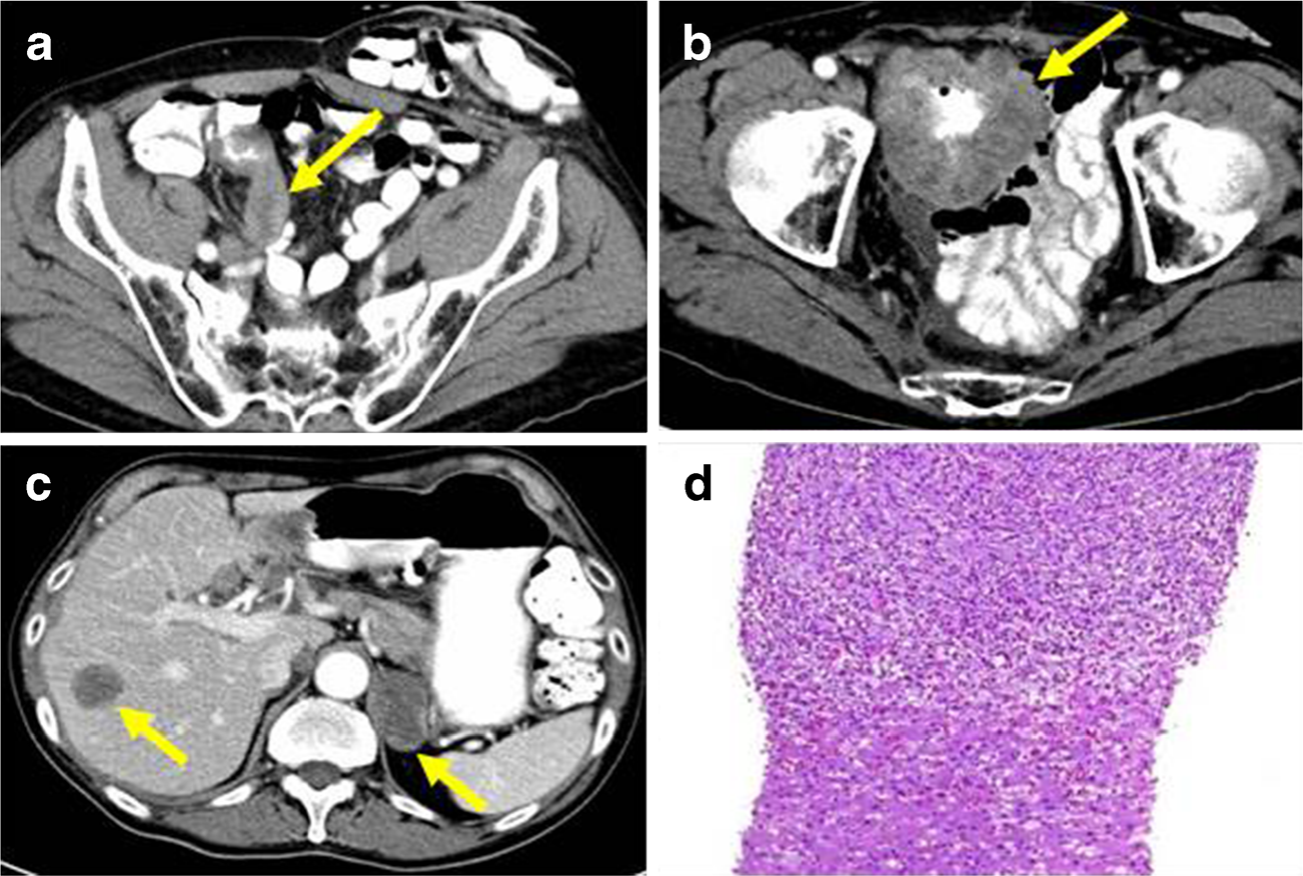
PATHOLOGY: metastatic IMT of the appendix. A 63-year-old man with antecedents of left hemicolectomy due to colon cancer. **a** Axial reformation of contrast-enhanced CT on portal phase acquired seven years after partial colectomy. An appendicular, irregular, solid mass with slight enhancement was identified (*yellow arrow*). Due to its radiological features, the lesion was diagnosed of appendicular carcinoma. The patient did not perform surgery. **b** Axial reformation of contrast-enhanced CT on portal phase performed 8 months later showed a significant growth of the appendicular mass with extension and infiltration of the adjacent small bowel loops. **c** Distant lymphadenopathy and liver metastases were present

Contrast-enhanced CT is the imaging technique, which give us more information about of IMTs of the gastrointestinal tract. They are usually found as hypodense, heterogeneous and well-delimited masses (Figs. 10, 11 and 12).

The differential diagnosis of these tumours includes GIST, inflammatory fibroid polyp, smooth muscle neoplasm, peripheral nerve sheath tumour, solitary fibrous tumour, fibromatosis, the follicular dendritic cell sarcoma, lymphoma and adenocarcinomas [9]. In most of these cases imaging features are not enough to diagnose IMTs and biopsy or surgery are needed.

Surgery is the most accepted treatment. IMTs of the gastrointestinal tract present a higher recurrence rate than in other locations of the body [29]. Other treatments, such as steroids, non-steroidal anti-inflammatory drugs and thalidomide, have been used in these tumours. Radiotherapy may have a place as adjunctive therapy in local recurrence and incomplete surgical removal [32].

IMTs of the appendix are extremely rare, with few cases reported in the literature [9, 33, 34]. Chronic infections, antecedents of trauma or surgery have been proposed as possible aetiological factors [9]. Associations with acute appendicitis [35] and pseudomyxoma [9] have been described.

Their radiological appearance is similar to the IMTs of other gastrointestinal locations. Histological confirmation is needed to achieve the diagnosis.

They usually have a benign clinical course with complete resolution after appendicectomy. Extremely rare cases of IMTs of the appendix showing local aggressiveness and metastases have been reported [36] (Fig. 12).

### Mesenteric IMTs

Mesentery is one of the most common locations of extrapulmonary IMTs. They are more frequent in children and young adults [37].

Many different aetiological factors have been proposed for mesenteric IMTs [37, 38], such as chronic infections, autoimmune diseases and trauma. They frequently appear in patients with previous surgery procedures [3, 39]. On contrast-enhanced CT they usually appear as solid masses with heterogeneous enhancement pattern and ill- or well-defined margins. Their most frequent clinical presentation is as an abdominal mass. They are often associated with a general inflammatory response manifested with fever, weight loss and other symptoms that are related to the compressive effect of the tumour, such as abdominal pain and vomiting.

Management of mesenteric IMTs is variable. Imaging, usually CT. Rapidly growing tumours or symptomatic ones needing treatment may follow from small tumours which are not encroaching any nearby structures. Different treatments have been proposed: non-steroidal anti-inflammatories, anti-oestrogens, chemotherapeutic agents and surgery. Surgery remains as a useful modality although quite a high local recurrence rate has been described for tumours [3, 37, 38].

### Musculoskeletal IMTs

**Fig. 13:**
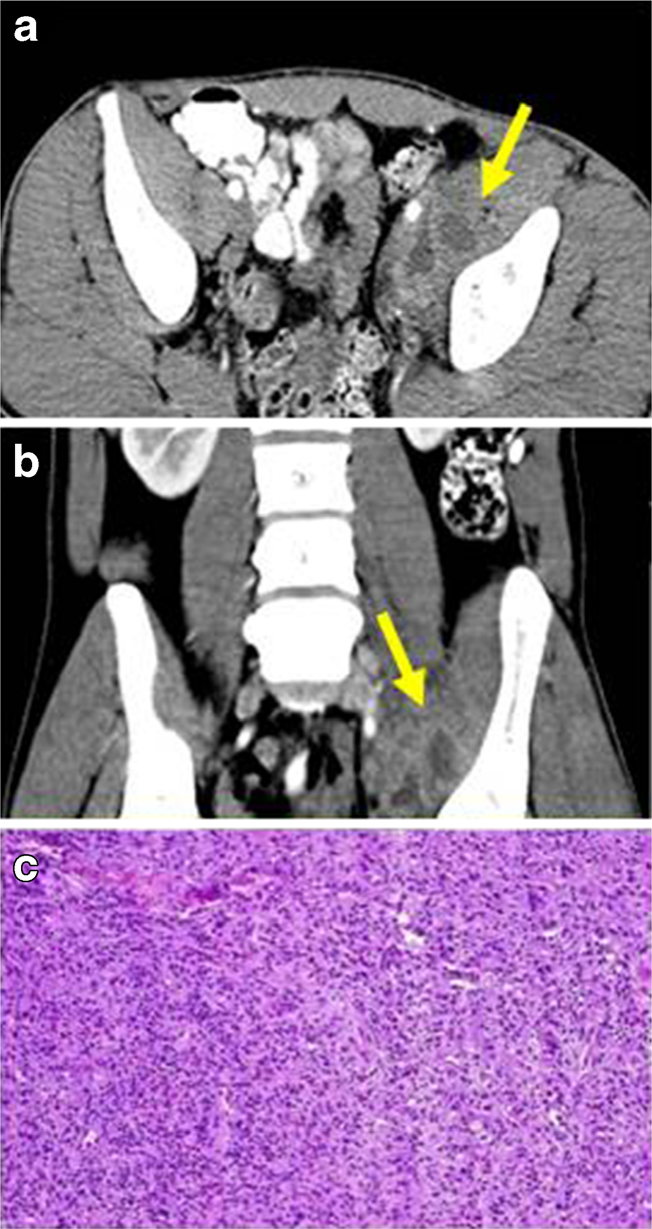
PATHOLOGY: IMT of the iliopsoas muscle. **a** Axial and **b** coronal reformations of contrast-enhanced CT where an ill-defined and heterogeneous mass encompassing iliopsoas muscle and external iliac vessels, mimicking malignant sarcomatous neoplasm was observed. **c** Sample of the lesion obtained after surgical excision of the mass. Proliferation of fibroblast with a diffuse infiltration of lymphocytes, eosinophils and macrophages were found. No mitotic figures or cellular atypia were present. The mass was well-defined and no signs of invasiveness were found

Few cases have been described of IMTs arising from the muscles [40]. Clinical manifestations usually are due to compression of neighbouring organs. The symptoms that most often appear include pain, haematuria, dysuria or urinary tract obstruction. The radiological features do not help to distinguish them from malignant neoplasms, such as rabdomyosarcomas (Fig. 13). Due to its deep location, fine-needle biopsy may fail to yield a sufficient volume of tumour tissue to achieve diagnosis.

## Conclusions

Although IMTs in some organs are not uncommon, they are not usually included in the differential diagnosis of nodules and masses. Their radiological features suggest malignant neoplasms, whereas they are not.

Consequently, this is an underdiagnosed entity and only after an histological exam could definitive diagnosis be achieved.

It is fundamental for radiologists to know about the existence of IMTs, as they frequently have better treatment options and prognosis than the malignant neoplasms they are confused with.
